# Ability emotional intelligence in young people and older adults with and without depressive symptoms, considering gender and educational level

**DOI:** 10.7717/peerj.6595

**Published:** 2019-04-19

**Authors:** Beatriz Navarro-Bravo, José M. Latorre, Ana Jiménez, Rosario Cabello, Pablo Fernández-Berrocal

**Affiliations:** 1 Department of Psychology, Universidad de Castilla La Mancha, Albacete, Spain; 2 Unidad de Investigación, Gerencia de Atención Integrada de Albacete, Fundación del Hospital Nacional de Parapléjicos, Albacete, Spain; 3 Universidad de Castilla La Mancha, Albacete, Spain; 4 Department of Developmental Psychology and Education, Universidad de Granada, Granada, Spain; 5 Department of Basic Psychology, Universidad de Málaga, Málaga, Spain

**Keywords:** Emotional intelligence, Elderly, Gender, Adulthood, Educational level, Depression

## Abstract

**Background:**

There is little research on differences in Emotional Intelligence (EI) ability at different stages of adult development. The few published studies tend not to use older adult samples. Previous studies on EI ability and age have shown contradictory results. Our main objective was to evaluate results in EI ability across different stages of adult development, taking into account gender, depressive symptoms, and educational level.

**Methods:**

We interviewed 166 participants (108 women), 66 of whom were aged 18–30 years, 53 aged 31–60 years, and 40 aged 61–76 years. All were either working or enrolled in colleges at the time of the study. The assessment tools used were the Mayer-Salovey-Caruso Emotional Intelligence Test (MSCEIT), a test that assesses performance-based EI, and the Centre for Epidemiologic Studies-Depression (CES-D) scale, a tool to evaluate depressive symptoms.

**Results:**

Young people, women, and participants with a higher educational level achieved higher scores on the MSCEIT. Additionally, depressive symptomatology was only partially associated with the MSCEIT (i.e., with the using emotions branch). However, a subsequent joint analysis of the independent effects of variables age, gender, educational level, and depressive symptomatology and their interactions on MSCEIT total suggests that only educational level and depressive symptomatology were associated with EI ability, with the direct relationship between age and gender with MSCEIT disappearing. Additionally, our study indicated an interaction effect between age and depressive symptoms, showing that participants in age cohorts 18–30 and 31–60 and without depressive symptoms have a higher EI ability.

**Discussion:**

Our study suggests that the direct effects of age and gender on EI ability across adult development, using a wide age range, can change or disappear when effects of educational level and depressive symptomatology, and their interactions, are controlled for. Our results also suggest that EI ability is a protective factor against depression in some age cohorts. This novel aspect of our study does not appear in the previous literature. However, prospective studies are needed to verify these findings and examine whether other psychological variables could determine the relations between age, gender and EI ability across adult development.

## Introduction

Currently, the most widely accepted definition of Emotional Intelligence (EI) is as a construct that “involves the ability to perceive accurately, appraise, and express emotion; the ability to access and/or generate feelings when they facilitate thought; the ability to understand emotion and emotional knowledge; and the ability to regulate emotions to promote emotional and intellectual growth” (Mayer & Salovey, 1997).

In recent years, many instruments have emerged to measure EI, which can be categorized into three main approaches (Joseph & Newman, 2010): performance-based ability model, self-report ability model and self-report mixed model.

Although all these approaches are used in EI research, the performance-based ability model has received greatest empirical support, providing better validity in predicting personal and life skills (Mayer, Caruso & Salovey, 2016; Megías et al., 2018; Webb et al., 2013). Ability or performance scales are based on the model of emotional processing of information, which proposes an assessment based on emotional tasks (Mayer et al., 2003). A participant’s responses are compared with the responses given by a large normative group (consensus criteria) or with the responses given by a group of recognized experts in the field of emotions (expert criteria). The advantages of these methods are the reductions in the effect of social desirability and the tendency to falsification, while the disadvantages are the difficulty in determining which answers are correct and the great amount of time needed for completion, with the possible effect of fatigue that this entails (Roberts, Schulze & MacCann, 2007). Additionally, self-report and performance measures of EI have been shown to be weakly correlated (Brackett et al., 2006).

There is little previous research available on differences in EI ability at different stages of adult development. Previous studies on EI ability and age have shown contradictory results. In a study using a sample of people between the ages of 19 and 66, it was found that older participants had somewhat higher scores on EI than younger ones (Kafetsios, 2004), including factors of using, understanding and managing emotions. Furthermore, in the study performed in Spain by Extremera, Fernández-Berrocal & Salovey (2006), it was found that scores on the Mayer-Salovey-Caruso Emotional Intelligence Test (MSCEIT) increased with age in a sample aged 16–58 years. In addition to age, higher scores on performance measures of EI are related to education and receiving psychotherapy (Goldenberg, Matheson & Mantler, 2006). A longitudinal study of women aged 65 and older found that emotional functioning improved during a 9-year follow-up period, from a median at baseline of 0.65 to a median of 0.78 nine years later (Zaslavsky et al., 2014).

However, other studies have found no significant relation between age and MSCEIT dimensions (Farrelly & Austin, 2007). Finally, some authors have even obtained a negative correlation between age and emotional perception ability (Cabello et al., 2014; Day & Carroll, 2004; Joseph & Newman, 2010; Palmer et al., 2005). In a recent study by Cabello et al. (2016) with a large sample of participants between 17 and 76 years, the authors found that EI ability using MSCEIT varied with age following an inverted-U curve. In general, younger and older participants scored lower on EI than middle-aged adults.

In another study comparing people with mild Alzheimer disease, aged normal controls and young normal controls, no differences were found in sensitivity between young and aged normal controls in the recognition of happiness in facial expressions. However, aged controls were less sensitive compared to young participants in recognition of surprise, anger and disgust (Maki et al., 2013). Regarding emotion recognition, West et al. (2012) found different results depending on the kind of emotion studied. Increased age was associated with a better recognition for the disgust expression, but the oldest age groups performed worse in the recognition of fear, anger and sad emotions in faces. With regard to other types of intelligence/dimensions of cognition, cognitive functions have been found to decline with age, including the speed of information processing and memory performance (Salthouse, Fristoe & Rhee, 1996; Bisiacchi et al., 2008; Johnson et al., 2009).

In relation to gender, the literature shows that EI ability differs in women and men (Brackett, Mayer & Warner, 2004; Joseph & Newman, 2010; Palmer et al., 2005). In the study validating the Spanish version of the MSCEIT score, the authors found that women scored higher than men in EI (Extremera, Fernández-Berrocal & Salovey, 2006). In a more recent study, with participants of a wide age range (17 to 76), the authors reported that EI ability was higher in women (Cabello et al., 2016).

The MSCEIT score has been found to correlate with other cognitive performance tests such as verbal subscales of IQ tests (*r* = 0.36) (Roberts, Schulze & MacCann, 2007) or the general intelligence test (Roberts, Zeidner & Matthews, 2001). It has been found across a wide range of studies that when large samples of people of different ages are compared using intelligence measures, the decrease in scores is much lower when the educational level is controlled for (although the differences do not disappear completely) (Kaufman, Reynolds & McLean, 1989). In recent years, it has also been found that fluid intelligence is related to education as strongly as crystallized intelligence is (Kaufman et al., 2009). If we focus on EI ability, there is evidence in the literature that educational history can protect against age-related EI decline by mediating the relationship between age and EI (Cabello et al., 2014). Specifically, using an additional moderation analysis, the authors showed that the EI scores of older adults with a university education were higher than those of older adults with primary or secondary education, and equivalent to those of younger adults of any education level.

EI is also associated with a number of health indicators such as depression or well-being (Cabello & Fernández-Berrocal, 2015; Zeidner, Matthews & Roberts, 2012). In a recent review, it was shown that better EI ability is negatively associated with depression (Fernández-Berrocal & Extremera, 2016). For example, Latorre et al. (2013), who performed an experimental procedure including the MSCEIT subtest of emotion perception in faces, found that recognition of a positive emotion in a neutral face is related to the level of depressive symptomatology. The authors found that participants with depressive symptoms evaluate a neutral face in a more negative way. Rode (2016) showed that EI, measured with the MSCEIT, predicts postpartum depression in addition to social support and stressful life events. A study by Sawaya et al. (2015) suggests that individuals with depression have reduced functional connectivity between antero-medial pre-frontal cortex (PFC) regions and regions involved in emotional regulation compared with control subjects. Moreover, ventromedial PFC functional connectivity appears to be linked to EI ability. However, the relation between EI ability and depression is moderated by several factors such as, for example, gender. It has been found that low levels of EI ability are related to higher depression in men, but not in women (Salguero, Extermera & Fernández-Berrocal, 2012).

Considering the contradictory results of previous research regarding EI ability and age and the fact that very few studies have measured EI ability in older adults, we have performed an exploratory study with the main objective of comparing the MSCEIT scores between young and old people but examining the join influence of gender, the educational level, depressive symptoms, and their interactions on EI scores. This objective is important because previous studies have analyzed the independent or the moderation and mediation effects of these different variables on EI ability in older adults (e.g.,  Cabello et al., 2014; Salguero, Extermera & Fernández-Berrocal, 2012), but not their joint and interaction effects.

Our starting hypotheses are four:

Firstly, taking into account the relationship between EI and other performance tests (Roberts, Zeidner & Matthews, 2001), we expect the MSCEIT score, as a type of intelligence, to show a similar decreasing pattern in adulthood (Johnson et al., 2009).

Secondly, with regard to gender, we predict the results will be consistent with previous studies showing women score significantly higher on EI ability than men (Cabello et al., 2016).

Thirdly, we consider that an individual’s educational level might influence their results in EI, with scores being higher in participants with a higher educational level (Cabello et al., 2014).

Finally, participants with depressive symptoms will score lower on EI ability (Fernández-Berrocal & Extremera, 2016).

## Method

### Design and variables

This is a cross-sectional observational study. The variables used were: gender, age, educational level, scores on the four factors assessed on the Mayer-Salovey-Caruso Emotional Intelligence Test V.2.0 (MSCEIT) (Perceiving, Using, Understanding and Managing emotions), the global score on the MSCEIT and depressive symptomatology measured using the Centre for Epidemiologic Studies-Depression (CES-D) scale.

#### Participants

This work was conducted with the voluntary collaboration of 166 participants ranging from 18 to 76 years old. The sample comprised 58 men and 108 women. Mean age was 42.75 years (SD = 19.28). The variable age was recoded in three age cohorts: age 18–30, 31–60, and 61–76. Specifically, 66 participants were 30 years old or less (59.1% women), 53 were between 31 and 60 years old (67.9% women), and 47 were 61 or older (70.2% women). The sample of participants up to age 60 were university students or teaching staff. The individuals of over 61 years were students from a university program for older adults. With respect to participants’ level of education, 4.8% had primary level studies, 19.3% high school, and 75.9% college education.

Inclusion criteria were: age (older than 18 years), working or studying at the time of this research and not having any physical or psychological disability that impeded their answering the questionnaires. We sought volunteers who were students or teachers at the time of the study as a way of ensuring they had the level of cognitive and intellectual activity required to complete the MSCEIT questionnaire.

#### Instruments

As a measure of EI, we used the most popular and well-established performance measure for assessing EI ability: the MSCEIT. Specifically, we used the Spanish version of the MSCEIT V.2.0 (Extremera, Fernández-Berrocal & Salovey, 2006; Mayer, Salovey & Caruso, 2002; Mayer et al., 2003). This questionnaire assesses EI using 141 items divided into eight skill sub-tests. With this test, it is possible to obtain scores that differ in their level of generality. This scale provides a global score of EI, and four sub-factors: perceiving emotions, using emotions, understanding emotions, and managing emotions. The reliability of the two halves is 0.93 for the consensus criterion and 0.91 for the criterion of experts. In the case of the four factors, the reliability of both methods is between 0.76 and 0.91 (Mayer et al., 2003). The test-retest reliability for the global MSCEIT at three weeks is 0.86 (Brackett & Mayer, 2003). We used the consensus criterion for this study, which compares participant’s responses with the responses given by a large normative group. The Spanish version has shown adequate psychometric properties in young, midlife, and older adults (Cronbach’s *α* = .95; Cabello et al., 2016;  Sánchez-Garcia, Extremera & Fernández-Berrocal, 2016). In our sample, Cronbach’s alpha was .89.

For depressive symptomatology, we used the Spanish translation of the Centre for Epidemiologic Studies-Depression (CES-D) scale (Radloff, 1977; Latorre & Montañés, 1997). It consists of 20 items that evaluate different aspects of depressive symptomatology such as depressed mood or hopelessness. It is widely used in research with adults and it has good reliability, internal consistency and discriminant and construct validity (Radloff, 1977; Radloff & Teri, 1986). We used a cut-off point of 16, widely used to detect possible depression (Weissman et al., 1997; Zunzunegui et al., 1998). It is also a valid instrument in older adults (Ros et al., 2011). In our sample, Cronbach’s alpha was .89.

### Procedure

The sample was selected from different educational institutions in the province of Albacete (Spain). Several teachers collaborated in the selection of volunteers from among their students, and also participated in the study. The questionnaires were self-administered in groups. We received verbal consent from the participants.

The data were entered into a database using the SPSS package (version 20.0; IBM, Armonk, NY, USA). Descriptive analyses of the sample were conducted and, to assess the fourth hypothesis, comparisons were made between age (one-way ANOVA), gender (Student t), education level (one-way ANOVA), and level of depression (Student t) groups for the scores on MSCEIT. To test the effect of all these variables on EI levels, a four-way ANOVA was performed. As dependent variable, we chose the total MSCEIT score. The independent variables were age, gender, educational level, depressive symptoms, and all second-order interactions. Third-order interactions were not estimated because we did not have the sample size needed to compute all the combinations.

With the aim of assessing the effect sizes of the ANOVA, we converted partial eta squared into Cohen’s D. It can be considered that from a threshold of 0.20 the effect is small, from 0.50 medium, and from 0.80 large (Cohen, 1988).

The study was approved by the Albacete Hospital Research Ethics Committee.

**Table 1:** MSCEIT and CES-D across age and gender groups.

	Participants grouped by age								Participants grouped by gender					
	18–30 years		31–60 years		61–76 years				Men		Women			
	(*n* = 66)		(*n* = 53)		(*n* = 47)				(*n* = 58)		(*n* = 108)			
Measure	M	SD	M	SD	M	SD	F	p	M	SD	M	SD	t	p
Emotion perception	0.47	0.09	0.47	0.10	0.43	0.13	2.03	.134	0.43	0.11	0.47	0.11	−2.20	.029
Emotion use	0.42	0.05	0.41	0.06	0.39	0.10	2.84	.061	0.40	0.7	0.41	0.07	−0.98	.330
Emotion understanding	0.46	0.05	0.45	0.06	0.42	0.07	6.04	.003	0.44	0.06	0.45	0.06	−0.67	.505
Emotion management	0.39	0.06	0.40	0.04	0.39	0.06	0.98	.376	0.38	0.06	0.40	0.05	−1.61	.110
Total score	0.44	0.05	0.43	0.05	0.41	0.06	4.23	.016	0.41	0.05	0.43	0.05	−2.07	.040
Depression (CES-D)	15.68	8.71	14.33	8.33	15.74	10.19	0.38	.682	12.17	7.52	16.93	9.27	−3.17	.002

**Table 2:** MSCEIT across educational level and depressive symptoms groups.

	Participants grouped by educational level								Participants grouped by depressive symptoms					
	Primary studies		High school		College education				No symptoms		Symptoms			
	(*n* = 8)		(*n* = 32)		(*n* = 126)				(*n* = 89)		(*n* = 58)			
Measure	M	SD	M	SD	M	SD	F	p	M	SD	M	SD	t	p
Emotion perception	0.41	0.16	0.42	0.13	0.47	0.09	4.14	.018	0.46	0.11	0.46	0.11	−0.16	.870
Emotion use	0.30	0.11	0.39	0.08	0.42	0.06	15.00	.000	0.42	0.06	0.39	0.08	1.99	.050
Emotion understanding	0.38	0.06	0.43	0.06	0.46	0.06	9.86	.000	0.46	0.06	0.44	0.07	1.41	.161
Emotion management	0.37	0.06	0.39	0.06	0.40	0.05	0.69	.501	0.40	0.05	0.39	0.05	1.00	.319
Total score	0.36	0.07	0.41	0.06	0.44	0.05	11.48	.000	0.43	0.05	0.42	0.06	1.26	.210

## Results

Descriptive statistics for the study variables are shown in Table 1 and Table 2. Regarding the first two hypotheses of this study, Table 1 shows the MSCEIT and CES-D scores for the groups arranged by age and gender. With regard to age, the one-way ANOVA revealed a main effect only for the MSCEIT total and the understanding branch. Post hoc analyses for the MSCEIT total and the understanding branch revealed that differences were found between age cohorts 18–30 and 61–76 (*p* = .02 for the MSCEIT total, and *p* = .002 for the understanding branch), and between ages 31–60 and 61–76 (*p* = .047 for the MSCEIT total, and *p* = .04 for the understanding branch), with age cohorts 18–30 and 31–60 attaining higher EI scores. For the other branches and for CES-D scores, no significant differences were found (Table 1).

With regard to gender, *t*-test analyses showed differences in the MSCEIT total and the perceiving branch, and in CES-D scores. In particular, women showed higher scores than men in the MSCEIT total and the perceiving branch, but also in CES-D scores (Table 1).

In relation to the third and fourth hypotheses of this study, Table 2 shows the MSCEIT scores for the groups arranged by educational level and depressive symptoms.

With respect to educational level, the one-way ANOVA revealed a main effect for the MSCEIT total and all EI branches, except management. Post hoc analyses revealed that differences were found between educational level primary studies and college education (*p* = .001 for the MSCEIT total, and the using emotions and understanding branches), between high school and college education (*p* = .006 for the MSCEIT total, *p* = .03 for the perceiving emotions, *p* = .026 for the using emotions, and *p* = .029 for understanding emotions branches), and between primary studies and high school (*p* = .003 for the using emotions branch)*,* with college education attaining higher EI scores (Table 2).

With regard to depression, *t*-test analyses showed differences only in the using emotions branch. In particular, participants with depressive symptoms showed lower scores in this EI dimension (Table 2).

**Figure 1:**
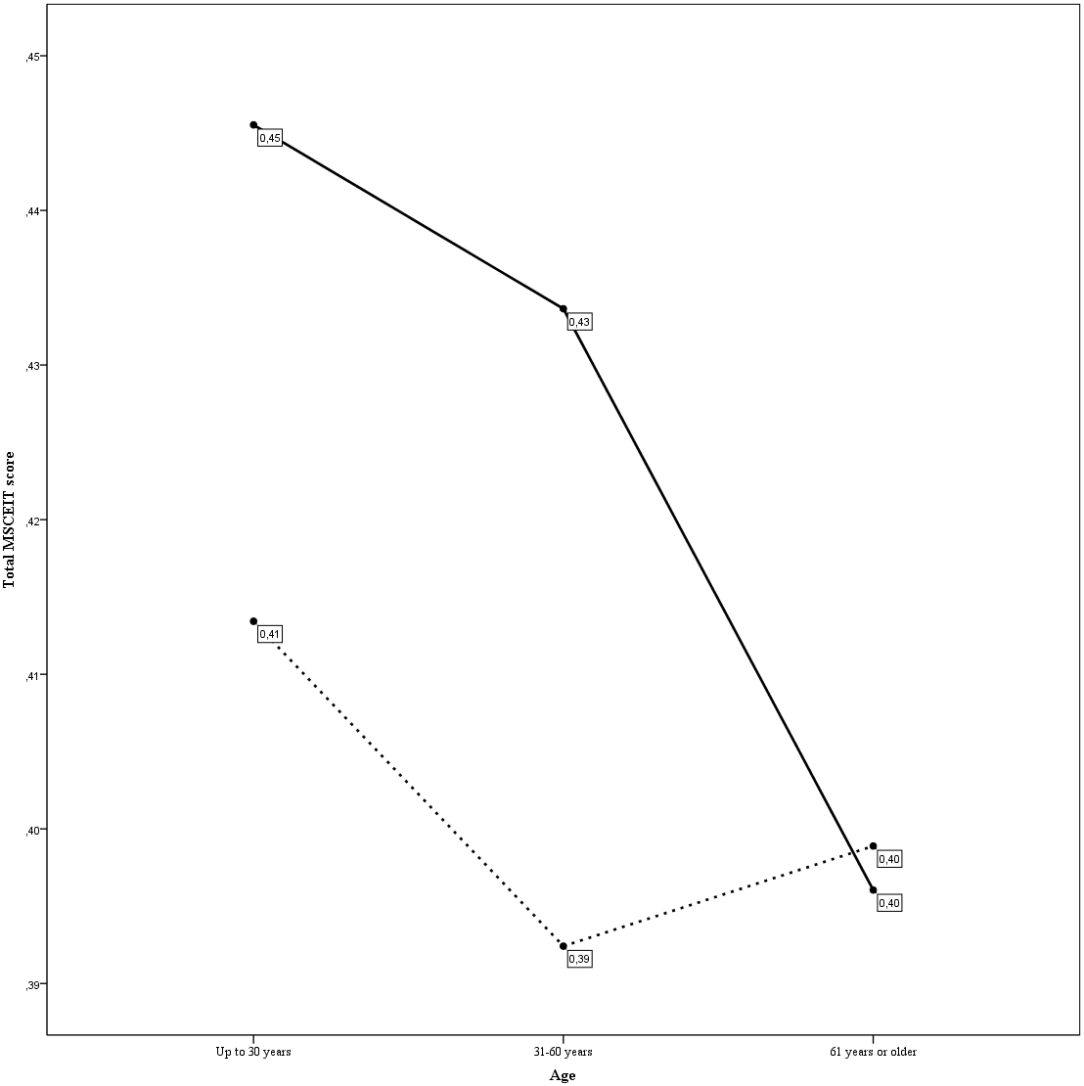
Interaction between age and depressive symptoms (CES-D cut off point equal to 16) in MSCEIT total score. The continuous line refers to participants without depressive symptomatology; the discontinuous line refers to participants with depressive symptomatology.

To compare the EI scores by age cohorts (three age groups), taking into account the joint effect of gender, educational level, depressive symptomatology and their interactions, we performed a four-way ANOVA with the MSCEIT total score as dependent variable. For this analysis, the MSCEIT total was chosen, as it is the score that best reflects the participants’ overall EI. The results revealed statistically significant differences by educational level (*F*(2, 129) = 5.11, *p* = .007, partial *η*2 = .073, *d* = 0.56), as we found in the previous one-way ANOVA, and depressive symptoms (*F*(1, 129) = 5.90, *p* = .017, partial *η*2= .044, *d* = 0.43). By contrast, for the age cohort and gender variables, no significant differences were found. Additionally, there was an interaction effect between age cohorts and depressive symptoms (*F*(2, 129) = 3.98, *p* = .021, partial *η*2 = .058, *d* = 0.50). All the effect sizes ranged between small and medium. In Fig. 1, we can see the graphic representation of this interaction. There were statistically significant differences between individuals with and without depressive symptoms in the 18–30 (*p* = .016) and 31–60 (*p* = .006) groups, but not for the 61–76 group (*p* = .895), showing that participants in age cohorts 18–30 and 31–60 and without depressive symptoms have the highest MSCEIT total scores.

## Discussion

The present study shows that an analysis of the independent effects of variables age, gender, educational level, and depressive symptomatology on EI ability indicates that young people, women, and participants with higher educational level achieve higher scores in the MSCEIT, and that depressive symptomatology is only partially associated with EI ability (i.e., with the using emotions branch). However, a subsequent joint analysis of the independent effects of variables age, gender, educational level, and depressive symptomatology and their interactions on the MSCEIT total score suggests that only educational level and depressive symptomatology are associated with EI ability, with the direct relationship between age and gender with EI ability disappearing. Additionally, our study indicates an interaction effect between age and depressive symptoms, showing that participants in age cohorts 18–30 and 31–60 and without depressive symptoms have a higher EI ability.

In agreement with our first hypothesis, analysis of the independent effects of the age variable on EI ability indicates that older participants (>60 years) have the lowest scores on MSCEIT total and the understanding emotions branch. These results partly contradict some of the existing literature, which finds a positive correlation between age and the MSCEIT branches (Fernández-Berrocal & Extremera, 2016; Goldenberg, Matheson & Mantler, 2006; Kafetsios, 2004). However, these findings are consistent with other studies using a broader age range which suggest that older adults show lower EI ability than younger adults, and that this drop may reflect age-related decline of cognitive function, as described for cognitive intelligences (Cabello et al., 2014; Cabello et al., 2016; Day & Carroll, 2004; Joseph & Newman, 2010; Palmer et al., 2005).

On the other hand, and in agreement with our second hypothesis, analysis of the independent effects of variables gender on ability found that women scored significantly higher than men in the MSCEIT total and the perceiving branch. These findings showing better performance in EI ability by women are in line with previous literature (Brackett, Mayer & Warner, 2004; Cabello et al., 2016; Extremera, Fernández-Berrocal & Salovey, 2006; Joseph & Newman, 2010; Palmer et al., 2005).

With respect to our third hypothesis, our results on the association between education level and EI coincide with the previous literature. It is worth noting that the results in the MSCEIT present correlations with other cognitive performance tests such as general or verbal intelligences (Roberts, Schulze & MacCann, 2007; Roberts, Zeidner & Matthews, 2001). This could be one reason why the results show better performance by participants with a higher educational level, as often happens in other performance scales, such as cognitive intelligence tests (Escorial et al., 2003).

With regard to the fourth hypothesis, our results indicate that depressive symptomatology is only partially associated with MSCEIT, specifically, only with the using emotions branch. These findings contrast with previous research finding an association between depression and EI ability, particularly with the branches of understanding and managing emotions (Fernández-Berrocal & Extremera, 2016; Latorre et al., 2013; Rode, 2016; Sawaya et al., 2015).

However, a subsequent joint analysis of the independent effects of these key variables and their interactions on total EI ability suggests that these direct associations can change when we consider the effects of third variables. In particular, our results revealed that the direct relationship between age and gender disappears with the MSCEIT total score. It is important to note that previous studies have analyzed the independent or the interaction effects of these different variables on EI ability in older adults (e.g., Cabello et al., 2014; Salguero, Extermera & Fernández-Berrocal, 2012), but not the joint effects of these key variables and their interactions. This is in line with recent research that suggests key variables such as educational history or implicit theories about EI may help explain the observed influence of gender and age on EI ability (Cabello et al., 2014; Cabello & Fernández-Berrocal, 2015). These findings should help avoid simplistic explanations of EI as being determined principally by sociodemographic variables such as gender or age.

Finally, our results suggest that the association between the presence of depressive symptoms and the MSCEIT total is moderated by age, showing that the association between depression and EI ability is significant only for those below 61 years of age, but not in the older group of our sample (61 or more years). This finding coincides with those in the literature showing that the relation between EI ability and depression is moderated by several factors such as, for example, gender (Salguero, Extermera & Fernández-Berrocal, 2012). This novel aspect of our study does not appear in the previous literature (Fernández-Berrocal & Extremera, 2016) but, nevertheless, it must be taken with caution because it is the result of exploratory research. For example, the relation between EI ability and depression in the older group (61 or more years) may also be moderated by other important factors that were not necessarily measured in the present study. Further work should examine whether other psychological variables such as stress, social support or wellbeing might determine the relation between EI ability and depression in this age cohort (Zeidner, Matthews & Roberts, 2012).

### Limitations and future research

The fact that participants were selected by requesting their voluntary cooperation may limit the generalizability of the results, since those who volunteered to participate might have certain qualities that differentiate them from those who did not.

Regarding the variables studied and the focus of the study (the EI), it would have been appropriate to select individuals with a more homogenous level of education. We attempted to address this issue by choosing older individuals who were studying a university program for adults at the time of data collection.

The sample size is somewhat reduced, presenting the limitation that some subgroups have very few participants (e.g., participants with a low level of education).

Otherwise, our cross-sectional study prevents us from excluding the possibility of causality in the opposite direction among variables studies, for example, between depression and EI. Especially, if we take into account that the effect sizes were between small and medium. Therefore, prospective studies are needed to verify our results suggesting that higher EI ability is a protective factor against depression in age cohorts 18–30 and 31–60.

Notwithstanding these limitations, our study provided evidence to suggest that the direct effects of age and gender on EI ability across adult development, using a wide age range, can change or even disappear when effects of educational level and depressive symptomatology, and their interactions are controlled for. Our findings also suggest that EI ability and depressive symptoms are associated, but only for participants between age cohorts 18–30 and 31–60.

As a future line of research, we propose to assess EI ability and cognitive intelligence using longitudinal methodology to monitor the change of both types of intelligences across ages and the relationship between these two constructs at the different stages of adult development, but including other relevant variables as, for example, educational level or depressive symptoms that can determine these direct relationships.

Finally, a recent systematic literature review suggests that education has a protective effect on general cognition in response to several brain burden measures (Chapko et al., 2018). Therefore, the reasons why educational history has a protective capacity on EI ability could be linked to its positive association with a better cognitive reserve (Cabello et al., 2014). A practical implication of these findings for future studies is that a window is opened to carry out EI preventive intervention programs in populations potentially at risk across the life-span (e.g., people with a low educational level), which will have an additionally positive effect on their mental health (e.g., depression and anxiety) and well-being.

##  Supplemental Information

10.7717/peerj.6595/supp-1 Supplemental Information 1 Database with all the variables used in the study

## Additional Information and Declarations

### Data Availability

The following information was supplied regarding data availability: The raw data are available in the Supplemental File.
